# Biogenic Silver Nanoparticle: In vitro antifungal activity against *Sporothrix brasiliensis*

**DOI:** 10.1007/s42770-026-02022-7

**Published:** 2026-07-13

**Authors:** Luciéle Pereira de Melo, Caroline Quintana Braga, Carolina dos Santos Bermann, Yasmin Dummer Ruas, Maria Isabel de Azevedo, Gustavo Ruas de Araújo, Renata Osório de Faria, Angelita Reis Gomes, Luciano Aparecido Panagio, Karen Almeida de Araújo, Lara Baccarin Ianiski, Sônia de Avila Botton, Daniela Isabel Brayer Pereira

**Affiliations:** 1 Laboratório de Micologia, Programa de Pós-Graduação em Microbiologia e Parasitologia, Instituto de Biologia, Departamento de Microbiologia e Parasitologia, Universidade Federal de Pelotas (UFPel), RS 96160-000 Prédio 18, Sala 14. Campus Universitário Capão do Leão, s/n°, Pelotas, Brazil; 2 Laboratório de Biologia Molecular e Micologia, Programa de Pós-Graduação em Ciência Animal, Departamento de Medicina Veterinária Preventiva, Universidade Federal de Minas Gerais (UFMG), Escola de Veterinária, Belo Horizonte, Brazil; 3 Centro de Diagnóstico e Pesquisa em Micologia Veterinária, Programa de Pós-Graduação em Veterinária, Departamento de Veterinária Preventiva, Faculdade de Veterinária, Universidade Federal de Pelotas (UFPel), Pelotas, RS Brazil; 4 Programa de Pós-graduação em Microbiologia, Departamento de Microbiologia, Universidade Estadual de Londrina (UEL), Londrina, PR Brazil; 5 Laboratório de Pesquisas Micológicas, Programa de Pós-Graduação em Ciências Farmacêuticas, Centro de Ciências da Saúde (CCS), Departamento de Microbiologia e Parasitologia, Universidade Federal de Santa Maria (UFSM), Programa de Pós-Graduação em Medicina Veterinária, Santa Maria, RS Brazil

**Keywords:** Sporotrichosis, Susceptibility, Nanotechnology, Fungus, Resistance

## Abstract

*Sporothrix brasiliensis* is the predominant etiological agent of feline sporotrichosis in Brazil, where zoonotic dissemination represents an escalating public health concern. Reports of therapeutic refractoriness and the emergence of itraconazole-resistant isolates underscore the pressing need for alternative antifungal strategies. This study evaluated in vitro antifungal activity of a biogenic silver nanoparticle against 24 Brazilian clinical isolates of *S. brasiliensis* (18 cats and 6 dogs). Broth microdilution assays revealed minimum inhibitory concentrations ranging from 0.47 to 3.75 µg/mL with fungicidal and fungistatic activity. Scanning electron microscopy of treated yeast cells demonstrated pronounced morphological alterations, including cell wall discontinuities, cavitations, surface roughness, and leakage of intracellular contents, confirming severe structural damage. These findings demonstrate that the biogenic silver nanoparticle evaluated exhibits antifungal activity against *S. brasiliensis* and may represent a promising therapeutic alternative for sporotrichosis. Nonetheless, further in vivo studies are needed to validate their safety and clinical applicability.

## Introduction

*Sporothrix brasiliensis* is the main etiological agent of feline sporotrichosis, with significant public health impact, particularly in Brazil, where the disease exhibits endemic patterns. Over the past decades, numerous zoonotic cases of *S. brasiliensis* have been reported in several regions of Brazil [1] and in other South American countries, including Chile, Argentina and Uruguay [2]. In addition, reports of infections have also been described in North America and Europe [2, 3].

The most common therapies for human and animal sporotrichosis include itraconazole, terbinafine, potassium iodide, and amphotericin B. However, these drugs are associated with significant adverse effects and limited distribution in certain tissues [4]. Furthermore, the clinical response of feline sporotrichosis depends on several factors, including lesion severity, the overall health status of the patient, and treatment discontinuation or abandonment frequently results in relapse and worsening of the condition, which can lead to death [5]. In addition to these therapeutic challenges, in recent years an increasing number of *S. brasiliensis* isolates resistant to itraconazole have been reported, contributing to treatment failures and recurrence of infection [5–8].

This context has driven research toward new compounds and/or repurposed drugs. Historically, metal ions such as copper, silver and mercury have been evaluated for their antimicrobial properties. In this regard, the incorporation of silver into nanoparticles represents a promising alternative for the development of new antimicrobial agents [9]. The production of silver nanoparticles (AgNP) is a fundamental aspect of nanotechnology, as their morphological, physicochemical and biological properties are influenced by the synthesis process, which may involve synthetic, physicochemical or green/biogenic methods [10, 11]. Biogenic nanoparticles offer advantages over those obtained by chemical methods, as they are generated through simple, cost-effective, high-yield, and environmentally sustainable processes [10, 11]. Among the various applications of biogenic silver nanoparticles (Bio-AgNP), their antimicrobial properties stand out, particularly against multidrug-resistant bacteria, oomycetes, as well as yeast and filamentous fungi [9]. Interestingly, Bonilla et al. [4] tested and demonstrated in vitro and in vivo efficacy of a chitosan-functionalized Bio-AgNP against only one reference strain of *S. brasiliensis* and *S. schenckii*.

In the present study, we propose to evaluate in vitro activity of a Bio-AgNP against clinical isolates of *S. brasiliensis* obtained of felines and canines from two important endemic regions for sporotrichosis of Brazil.

## Materials and methods

Twenty-four Brazilian clinical isolates of *S. brasiliensis* obtained from felines (*n* = 18) and canines (*n* = 6) from Rio Grande do Sul and Minas Gerais states were included in this study (Table 1). All isolates were molecularly identified by amplification and sequencing of the calmodulin (CAL) gene, as previously described by Rodrigues et al. [12]. The Bio-AgNP used in the present study, designated NanoVerdeAg^®^, was obtained from GRAL Bioativos^®^ (Londrina, Brazil). This Bio-AgNP was synthesized through the reduction of AgNO_3_ using an aqueous extract from *Trichilia catigua* (catuaba) bark. The biosynthesis and subsequent characterization were performed in accordance with the patented methodology (BR1020210163755). The bioreduction of silver ions in the aqueous solution was monitored by sampling aliquots and recording the UV–Vis spectra. The absorbance of the synthesized AgNP was measured across a range of wavelengths, exhibiting a characteristic surface plasmon resonance peak at 450 nm. The resulting Bio-AgNP displayed an average hydrodynamic diameter of 100 nm, a zeta potential of − 23.27 mV, and a polydispersity index (PDI) of 0.17, at a final concentration of 10 nM. Although silver nanoparticles are prone to aggregation in sodium chloride-containing media, such as RPMI, the addition of the aqueous Bio-AgNP suspension to each well results in a two-fold dilution of the medium, thereby substantially reducing the likelihood of nanoparticle aggregation.

**Table 1:** Origin and in vitro susceptibility profiles of *Sporothrix brasiliensis* clinical isolates (*n* = 24) to biogenic silver nanoparticles (Bio AgNP)

Isolate^1^	Host	Geographic source		Bio-AgNP		Activity
		State^2^	Municipality	(µg/mL)		
				MIC^3^	MFC^4^	
MG 1	Cat	MG^1.1^	Belo Horizonte	0.93	3.75	Fungistatic
MG 6	Cat		Belo Horizonte	0.93	0.93	Fungicidal
MG 10	Cat		Belo Horizonte	3.75	7.5	Fungicidal
MG 13	Cat		Belo Horizonte	0.93	0.93	Fungicidal
MG 19	Cat		Belo Horizonte	0.93	0.93	Fungicidal
MG 21	Cat		Belo Horizonte	1.87	7.5	Fungistatic
RS 2	Cat	RS^1.2^	Capão do Leão	0.93	1.87	Fungicidal
RS 5	Cat		Capão do Leão	1.87	1.87	Fungicidal
RS 10	Cat		Capão do Leão	0.47	0.47	Fungicidal
RS 14	Cat		Capão do Leão	1.87	1.87	Fungicidal
RS 16	Cat		Capão do Leão	3.75	3.75	Fungicidal
RS 20	Cat		Capão do Leão	0.93	0.93	Fungicidal
RS 21	Cat		Capão do Leão	1.87	7.5	Fungistatic
RS 24	Cat		Capão do Leão	0.93	3.75	Fungistatic
RS 26	Cat		Capão do Leão	0.93	3.75	Fungistatic
RS 31	Cat		Capão do Leão	1.87	7.5	Fungistatic
RS 38	Cat		Capão do Leão	3.75	15	Fungistatic
RS 4615	Cat		Santa Vitória do Palmar	0.93	0.93	Fungicidal
RS 3	Dog		Pelotas	1.87	7.5	Fungistatic
RS 6	Dog		Pelotas	3.75	15	Fungistatic
RS 11	Dog		Pelotas	0.47	0.93	Fungicidal
RS 12	Dog		Pelotas	0.47	3.75	Fungistatic
RS 13	Dog		Pelotas	0.93	0.93	Fungicidal
RS 4612	Dog		Pelotas	1.87	7.5	Fungistatic
			MIC range (***µ*g/mL)**	**0.47–3.75**		
			GM^4^ **(*µ*g/mL)**	**1.32**		

^1^SisGen (*Sistema Nacional de Gestão do Patrimônio Genético e do Conhecimento Tradicional Associado*) registration numbers: ^1.1^A85C275 and ^1.2^AED6C29. ^2^ Brazilian State of MG: Minas Gerais and RS: Rio Grande do Sul. ^3^Minimum inhibitory concentration. ^4^Minimum fungicidal concentration. ^4^Geometric mean

Fungal inoculum preparation and broth microdilution assays were performed according to the Clinical and Laboratory Standards Institute document M27-A3 and methodology previously described [13]. Briefly, the inoculum was prepared from yeast-phase colonies of *S. brasiliensis* grown on Brain Heart Infusion (BHI) agar at 37 °C for 7 days. After yeast growth, the plates were flooded with 3 mL of sterile 0.85% saline solution, to which 0.01 mL of 20% Tween 20 was added. Colonies were scraped and the solution transferred to a tube, and homogenized by vortexing for 10 s. The supernatant was then transferred to a new tube and adjusted to a transmittance of 80%–82% at 530 nm. The yeast inoculum was diluted 1:50 in RPMI 1640 medium buffered with MOPS, resulting in a final concentration of 5 × 10² to 2.5 × 10³ CFU/mL. The microdilution assays were performed in 96-well polystyrene plates, with 100 µL of RPMI per well. Serial dilutions of Bio-AgNP, ranging from 120 µg/mL to 0.234 µg/mL, were prepared, followed by the addition of 100 µL of yeast inoculum to each well. Positive control (100 µL yeast inoculum + 100 µL RPMI) and a negative control (100 µL Bio-AgNP + 100 µL RPMI) were included in each assay. Plates were incubated at 37 °C for 72 h. Growth was visually assessed by comparing wells with the positive control. The minimum inhibitory concentration (MIC) was defined as the lowest concentration of the Bio-AgNP that completely inhibited the growth of *S. brasiliensis*. The MIC and two concentrations above of the MIC values were used to interpret the minimum fungicidal concentration (MFC). For this purpose, 10 µL aliquots from wells without visible fungal growth were plated onto Sabouraud dextrose agar (SDA) and incubated at 37 °C for 72 h. The MFC was interpreted as the lowest concentration of the compound that completely inhibited yeast growth. Fungicidal and fungistatic activities of Bio-AgNP were interpreted using the MFC: MIC ratio, where the Bio-AgNP was considered fungicidal when the ratio was 1 or 2, and fungistatic if the ratio was greater than 2 [13, 14]. All experiments were performed in quadruplicate.

Morphological alterations induced by Bio-AgNP on a clinical isolate of *S. brasiliensis* were evaluated by scanning electron microscopy (SEM) following a protocol formerly described [7], with modifications. In brief, after in vitro susceptibility test, yeasts were collected from wells with sublethal concentrations (Bio-AgNP concentration at which fungal growth occurred immediately above the MIC; 0.93 µg/mL) and from untreated control wells. Samples were centrifuged at 2000 rpm for 10 min, the supernatants were discarded, and the yeast pellets were fixed in 2.5% glutaraldehyde at 4 °C for 24 h. The pellets were then washed three times with PBS (pH 7.0), with centrifugation after each wash, and dehydrated in graded ethanol baths (30, 50, 70, 95, and 100%; 2000 µL each), followed by centrifugation at 2000 rpm for 30 min at each step. Finally, the pellets were dried in an oven at 37 °C for 72 h, mounted on stubs, sputter-coated with gold–palladium (60 s, 1.8 mM, 2.4 kV), and examined at 15 kV under magnifications ranging from 7000× to 25,000×.

## Results

**Fig. 1:**
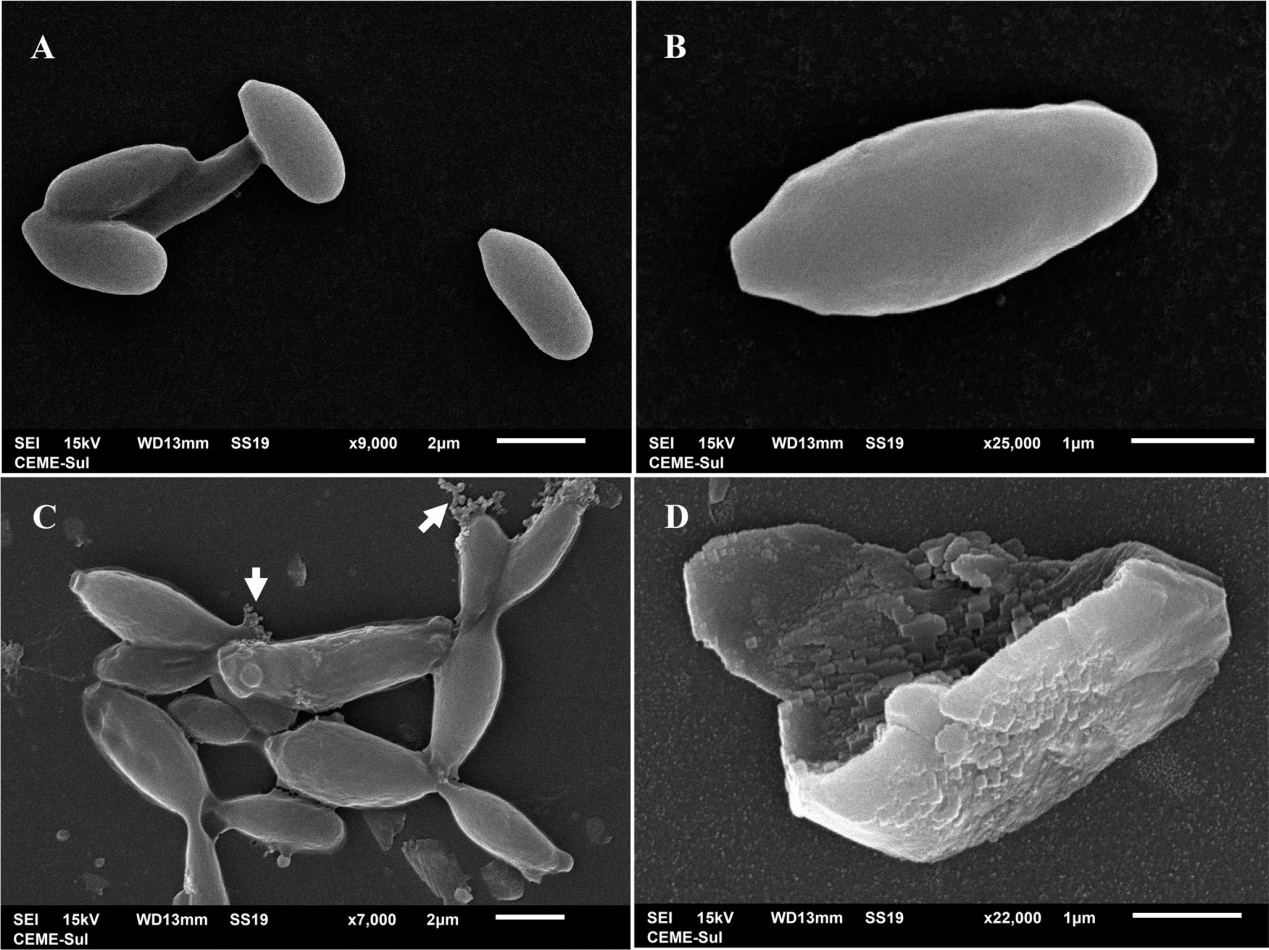
SEM images of *Sporothrix brasiliensis* yeast cells treated and untreated with Bio-AgNP. (**A** and **B**) Untreated control cells, showing typical yeast morphology, turgid appearance, and smooth, homogeneous surfaces without structural alterations. (**C** and **D**) Bio-AgNP-treated cells. (**C**) Pronounced morphological changes with dehydrated and shrunken appearance, rough and irregular surfaces and amorphous granular-like material at the cell extremities (arrow). (**D**) Cell wall discontinuities, cavitations and leakage of intracellular contents

Bio-AgNP inhibited in vitro growth of *S. brasiliensis*, with MIC values ranging from 3.75 to 0.47 µg/mL and a geometric mean (GM) of 1.32 µg/mL (Table 1). A fungicidal profile of the biogenic nanoparticle was observed in 54.17% (13/24) of the isolates, whereas a fungistatic profile was evidenced in 45.83% (11/24) (Table 1). SEM images of *S. brasiliensis* yeast cells treated and untreated with Bio-AgNP are shown in Fig. 1. Untreated control cells exhibited the typical morphology of yeast, appearing turgid with a smooth and homogeneous surface, without evidence of structural alterations (Fig. 1-A and 1-B). In contrast, Bio-AgNP treated cells displayed pronounced morphological changes, including a dehydrated and shrunken appearance, with rough and irregular surfaces (Fig. 1C). At the extremities of some cells, an amorphous, granular-like substance was observed (Fig. 1C, arrow). Additionally, some yeasts exhibited cell wall discontinuities, cavitations, and leakage of intracellular contents (Fig. 1D), indicating severe structural damage and potential cell lysis induced by Bio-AgNP.

## Discussion

Silver nanoparticles possess recognized antimicrobial potential and have been applied in various fields, including healthcare, food industry, water purification, clothing, and cosmetics [10, 11]. In the pharmaceutical industry, AgNP are commonly used in the form of creams or silver nitrate solutions for the treatment of burns, wounds and ulcers [15].

In this context, the present study evaluated in vitro antifungal activity of the biogenic silver nanoparticle NanoVerdeAg^®^ against clinical isolates of *S. brasiliensis* recovered from cats and dogs in two major endemic regions for sporotrichosis in Brazil. The Bio-AgNP evaluated demonstrated notable antifungal potential, as well as prominent structural damage in treated yeast cells.

In our study, Bio-AgNP inhibited the growth of *S. brasiliensis* with MIC values ranging from 0.47 to 3.75 µg/mL. These findings demonstrate relevant antifungal activity, particularly when compared with the data reported by Mathias et al. [16], who evaluated non-nanoparticulate silver salts (Ag-HPA) against *Sporothrix* spp. and reported substantially higher MIC values, ranging from 8 to 128 µg/mL. This difference suggests that silver nano-structuring enhances antifungal efficacy, due to the increased surface area-to-volume ratio and heightened reactivity, allowing efficient interactions with biomolecules located within or on the cell surface [10]. Likewise, our results corroborate those of Bonilla et al. [4], who investigated a chitosan-functionalized silver nanocomposite against a solely reference strain of *S. brasiliensis* and one strain of *S. schenckii*, observing inhibitory concentrations between 0.12 and 1 µg/mL. The MIC values obtained in our study were slightly higher, which may be attributed to the broader set of clinical *S. brasiliensis* isolates analyzed, reflecting greater phenotypic variability. Nevertheless, both studies reinforce the high potential of silver-based nanomaterials against *Sporothrix* spp.

Previous research demonstrated that the Bio-AgNP evaluated in the present study, either alone or in combination, exhibited no cytotoxic effects against HaCaT human keratinocytes, as assessed by the MTT cell viability assay. Likewise, no toxicity was observed in the alternative in vivo model *Galleria mellonella* [17].

Additionally, Bonilla et al. [4] demonstrated in vivo therapeutic efficacy, evidenced by the reduction of subcutaneous infection in a murine model of sporotrichosis and the promotion of tissue repair following topical treatment with a nanocomposite. Interestingly, the antimicrobial activity of AgNP has been reported not only against eukaryotes of the fungal kingdom [4, 11, 18–20] but also against mammalian pathogenic oomycetes [21], as well as Gram-positive and Gram-negative bacteria, including multidrug-resistant strains [22, 23]. Silver nanocomposites act on the fungal cell membrane, causing pore openings that compromise the membrane potential, ultimately leading to apoptosis [19]. Additionally, studies reported that apoptosis results from mitochondrial dysfunction and a consequent increase in oxidative stress, with the generation of reactive oxygen species (ROS) [18, 24]. In *Candida albicans*, the treatment with Bio-AgNP increased ROS production, promoting membrane destabilization and inhibiting yeast budding [9, 20]. Bonilla et al. [4] suggested that the antifungal activity of AgNP is due to interactions with the fungal cell wall and membrane, which induce structural alterations. These disturbances compromise cell permeability, leading to cytoplasmic leakage, lysis and cell death. In the present study, evident structural disturbances such as cellular deformities, surface roughness, discontinuities and rupture of the cell wall, and cavitations with intracellular content leakage, were observed in SEM images of *S. brasiliensis* cells treated with Bio-AgNP (Fig. 1C and D). These findings confirm the antifungal activity of the evaluated nanocomposite. Similarly, Mathias et al. [16] reported that *Sporothrix* spp. cells treated with non-nanoparticulate silver salts exhibited surface deformities, appearing corrugated and wrinkled. Furthermore, in a previous study evaluating the anti-*Pythium insidiosum* activity of a Bio-AgNP, Valente et al. [21] described surface roughness, retractions, and discontinuities in the cell wall of hyphae treated, consistent with our observations.

Previously, in vitro susceptibility of the same *S. brasiliensis* clinical isolates used in the present study was monitored to itraconazole [13]. Although all isolates behaved as wild type (susceptible) to the antifungal, the authors reported that 52% exhibited a MIC of 2 µg/mL, suggesting a trend toward reduced susceptibility to itraconazole. However, other in vitro studies conducted in Brazil have reported an increasing number of non-wild type (reduced susceptibility) *S. brasiliensis* isolates to itraconazole [6–8]. Thus, research aimed at identifying compounds with promising antifungal activity is both timely and necessary. In this context, the Bio-AgNP tested herein, a nanocomposite produced through sustainable and environmentally friendly technology, demonstrated excellent inhibitory activity against *S. brasiliensis*.

## Conclusion

The Bio-AgNP evaluated in this study exhibited both fungicidal and fungistatic in vitro activity against clinical isolates of *S. brasiliensis*. These findings support further investigation of this nanocomposite as a potential antifungal agent. However, additional studies addressing its mechanism of action, safety profile, pharmacokinetics, and efficacy in vivo are required before considering any therapeutic application.

## References

[1] Rabello VBS et al (2022) Environmental isolation of Sporothrix brasiliensis in an area with recurrent feline sporotrichosis cases. Front Cell Infect Microbiol 12:894297. 10.3389/fcimb.2022.894297 35646737 10.3389/fcimb.2022.894297 PMC9134204

[2] Xavier MO et al (2023) Sporothrix brasiliensis: epidemiology, therapy, and recent developments. J Fungi (Basel) 9(9):921. 10.3390/jof9090921 37755029 10.3390/jof9090921 PMC10532502

[3] 3.Poester VR et al (2024) Sporothrix brasiliensis causing atypical sporotrichosis in Brazil: a systematic review. J Fungi (Basel) 10(4):287. 10.3390/jof10040287 38667958 10.3390/jof10040287 PMC11051268

[4] Bonilla JJA et al (2022) Silver chitosan nanocomposites are effective to combat sporotrichosis. Front Nanotechnol 4:857681. 10.3389/fnano.2022.857681

[5] Gremião IDF et al (2021) Guideline for the management of feline sporotrichosis caused by Sporothrix brasiliensis and literature revision. Braz J Microbiol 52(1):107–124. 10.1007/s42770-020-00365-3 32990922 10.1007/s42770-020-00365-3 PMC7966609

[6] Nakasu CCT et al (2021) Feline sporotrichosis: a case series of itraconazole-resistant Sporothrix brasiliensis infection. Braz J Microbiol 52(1):163–171 10.1007/s42770-020-00290-5 32388779 10.1007/s42770-020-00290-5 PMC7966689

[7] Borba-Santos LP, Vila T, Rozental S (2020) Identification of two potential inhibitors of Sporothrix brasiliensis and Sporothrix schenckii in the pathogen Box collection. PLoS One. 2020; 15(10): e0240658. 10.1371/journal.pone.0240658 10.1371/journal.pone.0240658 PMC7556523 33052959

[8] Santos AR et al (2024) Emergence of zoonotic sporotrichosis in Brazil: a genomic epidemiology study. Lancet Microbe 5(3):e282–e290. 10.1016/S2666-5247(23)00364-6 38432234 10.1016/S2666-5247(23)00364-6 PMC11487493

[9] Tończyk A et al (2025) Mycogenic silver nanoparticles: promising antimicrobials with fungistatic properties. Int J Mol Sci 26(14):6639. 10.3390/ijms26146639 40724889 10.3390/ijms26146639 PMC12294507

[10] Mir RH et al (2024) Green Synthesis of silver nanoparticles and their potential applications in mitigating cancer. Curr Pharm Des 30(31):2445–2467. 10.2174/0113816128291705240428060456 38726783 10.2174/0113816128291705240428060456

[11] 11.Siddiqi KS, Husen A, Rao RAK (2018) A review on biosynthesis of silver nanoparticles and their biocidal properties. J Nanobiotechnol 16(1):14. 10.1186/s12951-018-0334-5 10.1186/s12951-018-0334-5 PMC5815253 29452593

[12] Rodrigues AM et al (2015) Rapid identification of emerging human-pathogenic Sporothrix species with rolling circle amplification. Front Microbiol 6:1385. 10.3389/fmicb.2015.01385 26696992 10.3389/fmicb.2015.01385 PMC4672047

[13] Melo LP et al (2025) In vitro susceptibility profile of Brazilian Sporothrix brasiliensis isolates to amorolfine hydrochloride and itraconazole. Med Mycol 63(9):myaf075. 10.1093/mmy/myaf075 40838978 10.1093/mmy/myaf075

[14] Ramos MLM et al (2024) In vitro activity of the anthelmintic drug niclosamide against Sporothrix spp. strains with distinct genetic and antifungal susceptibility backgrounds. Braz J Microbiol 55(2):1359–1368. 10.1007/s42770-024-01301-5 38466550 10.1007/s42770-024-01301-5 PMC11153390

[15] Essa MS, Ahmad KS, Zayed ME, Ibrahim SG (2023) Comparative study between Silver Nanoparticles Dressing (SilvrSTAT Gel) and conventional dressing in diabetic foot ulcer healing: a prospective randomized study. Int J Low Extrem Wounds 22(1):48–55. 10.1177/1534734620988217 33686887 10.1177/1534734620988217

[16] Mathias LS et al (2020) Antifungal activity of Silver Salts of Keggin-Type Heteropolyacids against Sporothrix spp. J Microbiol Biotechnol 30(4):540–555. 10.4014/jmb.1907.07064 31893614 10.4014/jmb.1907.07064 PMC9728368

[17] Castro IM et al (2025) Synergistic antibacterial interaction of Geraniol and Biogenic Silver Nanoparticles on Methicillin-Resistant Staphylococcus aureus. Plants 2025, 14: 1059. 10.3390/plants14071059 40219128 10.3390/plants14071059 PMC11991589

[18] Mussin J, Giusiano G (2022) Biogenic silver nanoparticles as antifungal agents. Front Chem 10:1023542. 10.3389/fchem.2022.1023542 36277355 10.3389/fchem.2022.1023542 PMC9583421

[19] Chandrakar N et al (2025) Biogenic silver nanoparticles exhibit antifungal and antibiofilm activity against Candida albicans via Intracellular ROS Production. APMIS 133(8):e70061. 10.1111/apm.70061 40823783 10.1111/apm.70061 PMC12358642

[20] Abdallah BM, Ali EM (2022) Therapeutic Effect of green synthesized silver nanoparticles using Erodium glaucophyllum extract against oral candidiasis: in vitro and in vivo study. Molecules 27(13):4221. 10.3390/molecules27134221 35807474 10.3390/molecules27134221 PMC9267989

[21] Valente JSS et al (2019) In vitro anti-Pythium insidiosum activity of biogenic silver nanoparticles. Med Mycol 57(7):858–863. 10.1093/mmy/myy147 30597067 10.1093/mmy/myy147

[22] Cunha KF et al (2023) Biogenic silver nanoparticles: in vitro activity against Staphylococcus aureus methicillin-resistant (MRSA) and multidrug-resistant coagulase-negative Staphylococcus (CoNS). Braz J Microbiol 54(4):2641–2650. 10.1007/s42770-023-01102-2 37676406 10.1007/s42770-023-01102-2 PMC10689704

[23] Allend SO et al (2022) Biogenic silver nanoparticle (Bio-AgNP) has an antibacterial effect against carbapenem-resistant Acinetobacter baumannii with synergism and additivity when combined with polymyxin B. J Appl Microbiol 132(2):1036–1047. 10.1111/jam.15297 34496109 10.1111/jam.15297

[24] Dakal TC, Kumar A, Majumdar RS, Yadav V (2016) Mechanistic basis of antimicrobial actions of Silver Nanoparticles. Front Microbiol. 27: 1831. 10.3389/fmicb.2016.01831 10.3389/fmicb.2016.01831 PMC5110546 27899918

